# 

**Published:** 1827-01

**Authors:** 


### THE MEDICO-CHIRURGICAL REVIEW.

*NEW SERIES.*

---

### VOLUME SIX.

---

[BEING VOL. X. OF ANALYTICAL SERIES.]

CONDUCTED BY

ASSOCIATED PHYSICIANS AND SURGEONS;

AND SUPERINTENDED BY

JAMES JOHNSON, M.D.

MEMBER OF THE ROYAL COLLEGE OF PHYSICIANS OF LONDON, AND PHYSICIAN EXTRAORDINARY TO HIS ROYAL HIGHNESS THE DUKE OF CLARENCE.

---

Nec tibi quid liceat sed quid fecisse decebit
Occurrat mentemque domat respectus honesti.—CLAUD.

---

Surgeon Gen'l's Office LIBRARY 5744

LONDON:

*Printed by G. Hayden, Little College Street, Westminster.*

Published by S. Highley, 174, Fleet Street, and Webb Street, St. Thomas's Hospital; Baldwin, Cradock, & Joy, Pater-Noster Row; Kingsbury, Parbury, & Allen, Leadenhall Street; T. & G. Underwood, Fleet Street; Callow and Wilson, Princes Street, Soho; Burgess & Hill, Windmill Street, Haymarket; Anderson, West Smithfield; Cox & Son, and Jackson, Borough; J. Nimmo, Great Maze Pond, Borough; Adam Black, Edinburgh; D. Lizars, Princes Street, Edinburgh; Richard Griffin & Co., W. R. M'Phun, and David Allan & Co. Glasgow; Hodges & M'Arthur, Dublin; Messrs. Treuttel & Wurtz, Paris and Strasburg.

---

1827.

### THE MEDICO-CHIRURGICAL REVIEW,

AND

### JOURNAL

OF

### PRACTICAL MEDICINE.

FIRST VOLUME,

[1st of JANUARY to 30th of JUNE,]

1827.

EDITED BY JAMES JOHNSON, M. D.

*&c. &c. &c.*

---

PUBLISHED BY S. HIGHLEY,

174, FLEET STREET,

AND WEBB STREET, ST. THOMAS'S HOSPITAL,

And all other Booksellers.

W1
ME75T

PRINTED BY G. HAYDEN,
Little College Street, Westminster.

### X.

### BIBLIOGRAPHICAL RECORD;

OR,

*Works received for Review between the 15th of September, and the 15th of December, 1826.*

1. The Philadelphia Journal of the Medical and Physical Sciences. No. 6, New Series, August, 1826. Philadelphia. Also, No. 7.

☞ *In exchange.*

---

2. A Lecture on the Uses of Anatomy and Physiology in various branches of knowledge, delivered on Monday, the 1st of November, 1824. By James Macartney, M.D. F.R.S. F.L.S. M.R.I.A. Professor of Anatomy and Surgery in the University of Dublin, &c. 8vo. stitched, pp. 36. Dublin, 1826.

---

3. The Edinburgh Journal of Medical Science, &c. No. IV. for October, 1826.

---

4. On Galvanism, with Observations on its Chemical Properties and Medical Efficacy in Chronic Diseases, with Practical Illustrations; also Remarks on some Auxiliary Remedies. With Plates. By M. La Beaume, Medical Galvanist, &c. &c. 12mo. pp. 271. London, 1826. Price 7s.

---

5. Principles of Medical Science and Practice. Part II. Vol. II. Pathology. By Hardwicke Shute, M.D. &c. &c. 8vo. pp. 604. London, 1826.

---

6. Disputatio Inauguralis de Colica-Biliosa Intertropica. Par Joannem Evans, M.D. F.R.C. S.L. &c.

☞ *We shall give some account of the disease with which the crew of His Majesty's Ship Bedford was scourged, in our Periscope.*

---

7. Outlines of Lectures on Mental Diseases. By Alexander Morison, M.D. of the Royal College of Physicians of London and Edinburgh, &c. &c. 8vo. pp. 128, with 14 plates, containing numerous figures. London, October, 1826. *Second edition.*

☞ *These Outlines contain many important observations on mental derangement, and the plates are beyond all praise, for the excellence of their execution (by Lizars) and the fidelity of the expression which they are designed to convey. We are happy to learn that Dr. Morison has taken up his residence in London, and means to continue his Lectures on Mental Diseases. We wish him every success.*

---

8. Manual of Pathology, containing the Symptoms, Diagnosis, and Morbid Characters of Diseases: together with an exposition of the different Methods of Examination, applicable to Affections of the Head, Chest and Abdomen. By L. Martinet, D.M.P. Resident Physician of the Hôtel Dieu. Translated, with Notes and Additions, by Jones Quain, A. B. Demonstrator of Anatomy at the Medical School, Aldersgate-street, London. 12mo, pp, 310, 1826. Price 6s.

☞ *This most excellent little work, we shall notice more at length, in another place.*

9. System of Anatomical Plates; with descriptive Letter-Press. By JOHN LIZARS, F.R.S. E. &c. &c. &c. Part XII. Gravid Uterus, and Lymphatics. Edinburgh. Price 10s. 6d. plain, or 1*l*. 1s. coloured after Nature.

☞ *This completes the Series, and the last plates are still better than the first. The unparalleled success of this undertaking proves the merit of all parties concerned—the drawer, engraver, and author. This system of plates is now such a standard production, that it would be superfluous to say more on the occasion. Mr. Lizars has gone on through an arduous undertaking, not merely "qualis ab incepto;" but he may safely say of the work—"viresque acquirit eundo."*

---

10. New-York Medical and Physical Journal, Nos. 17 and 18, for the first and second quarters of 1826.

☞ *In exchange.*

---

11. The Use of the Chlorate of Soda, and the Chlorate of Lime. By A. G. Labarraque, Pharmacien of Paris, &c. &c. Translated by JAMES SCOTT, Surgeon. London, 8vo, stiched, pp. 36, 1826. Price, 1s. 6d.

---

12. An Oration delivered before the Medico-Botanical Society of London, at the commencement of their Seventh Session, Friday, 13th October, 1826. By JOHN FROST, F.A.S. F.L.S. &c. &c. &c.

☞ *The Medico-Botanical Society, Sir James M'Gregor in the Chair, passed a vote of thanks to Mr. Frost, for his Oration, and requested that it might be printed. This is saying more than any eulogy which we could pass. We are glad to find that more attention is now paid to Medical Botany than formerly; and we hope Mr. Frost will continue his zealous labours in behalf of so delightful a science.*

---

13. Remarks on Phylolacea Dodecandron; or the Mustard-Tree of the Scriptures. By JOHN FROST, F.A.S. F.L.S. &c.

☞ *We understand the Bishop of London has written a letter to Mr. F. expressing his satisfaction at the elucidation thus given to a most difficult passage in Scripture.*

---

14. An Essay on Morbid Sensibility of the Stomach and Bowels, &c. By JAMES JOHNSON, M.D. &c. Octavo, pp. 132, price 5s. boards. Underwoods, 1827.

---

15. Obstetric Plate, No. 1. This Plate represents a Fœtus in Utero, enveloped in the Membranes at the 7½ Month of Gestation, with the Placenta attached to the Body, Cervix, and Os Uteri, as taken from the subject 36 hours after death. By GEORGE JEWEL, Surgeon, Lecturer on Midwifery, &c. Sold by Callow and Wilson, London.

☞ *This plate conveys an accurate idea of the partial separation of the Placenta. The unfortunate woman appears to have died of uterine hœmorrhage, no delivery having been attempted. Mr. Jewel did not see the patient while living.*

---

16. A Letter from George Combe, Esq. to Francis Jeffrey, Esq. in Answer to his Criticism on Phrenology, contained in the 88th Number of the Edinburgh Review. 8vo, pp. 78. John Anderson, Ed. Longman and Co. London, Nov. 1826.

☞ *The redoubtable Jeffrey has ventured beyond his depth, and completely committed himself. It is abundantly evident that he is ignorant of phrenology, and what is more, of anatomy and physiology, on which so much of the science of phrenology rests. Mr. Combe has given the anti-phrenologist a good dressing.*

---

17. Materia Indica; or, some Account of those Articles which are employed by the Hindoos, and other Eastern Nations, in their Medicine, Arts, and Agriculture; comprising also Formulæ, with practical Observations, Names of Diseases in various Eastern Languages, and a copious List of Oriental Books immediately connected with general Science, &c. By Whitelaw Ainslie, M.D. M.R.A.S. late of the Medical Staff of Southern India. Two volumes, 8vo, pp. 654 and 604. Price 40 shillings. Longman and Co. November, 1826.

☞ *Of these valuable and interesting volumes we shall give an extended analysis in our next number.*

---

18. The North American Medical and Surgical Journal. No. IV. October, 1826. ☞ *In Exchange.*

---

19. Observations upon the Autumnal Fevers of Savannah. By W. C. Daniel, M.D. 8vo, pp. 152. Savannah, 1826.

---

20. An Essay on the Use of Atropa Belladonna, or Solanum Lethale, and the Solanum Hortense, with practical Observations on their Effects in the Cure of Scirrhus, Cancer, Stricture, and various other Complaints. By Powel Charles Blackett, Member of the Royal College of Surgeons, &c. Octavo, pp. 68. Callow and Wilson, December, 1826.

---

21. Observations on the Utility and Administration of Purgative Medicines in several Diseases. By James Hamilton, M.D. Fellow of the Royal College of Physicians of Edinburgh, &c. Eighth Edition, revised and improved by the Author, with a Chapter on Cold Bathing, considered in its Purgative Effect. Octavo, pp. 202. Edinburgh and London, 1826.

---

22. An Introductory Lecture on Human and Comparative Physiology, delivered at the New Medical School in Aldersgate Street. By Peter M. Roget, M.D. F.R.S. &c. 8vo, pp. 103. Longman's London) 1826.

---

23. An Elementary Description of the Anatomy and Physiology of the Brain, Viscera of the Thorax, Abdomen, &c. with corresponding Questions. Designed for the Use of Gentlemen preparing for Examination at Apothecaries' Hall, and for Junior Students. By W. Simpson, F.R.C.S. &c. Second Edition, 12mo, stitched, pp. 55. London, 1826. Price 2s.

---

24. An Introductory Lecture on Anatomy, delivered at the New Medical School, Aldersgate Street, October 2d, 1826. By Frederick Tyrrell, Surgeon to St. Thomas's Hospital, &c. 8vo, pp. 74. Longman's London, 1826.

---

25. Additamenta ad Origines Contagii. Auctore C. F. H. Marx, Dr. Prof. Med. Gottingensi. Carolirhnæ et Baduæ. Apud D. R. Marx, 1826.

*This, like Dr. Marx's former work, of which we gave some account, contains a great mass of erudition and research.*

---

26. De Euthanasiâ Medicâ Prolusio, qua ad Orationem, quam ad Auspicandum Professoris Munus extra ordinem indulgentissime sibi concessum Die xxix. July mdcccxxvi. Publice habebit, rite invitat C. F. H. Marx, Med. et Chir. Dr. Gottingæ, Typis Dieterichianis expressum.

☞ *We return our thanks to the learned professor for his eloquent oration on the subject of euthanasia.*

---

27. Discussion Medico-legale, sur la Folie, ou Alienation Mentale, suivie de l'Examen du Procès Criminel d'Henriette Cornier, et de plusieurs autres Procès dans lesquels cette Maladie a été alleguée comme moyen de Défense. Par le Docteur Georget, &c. &c. &c. Octavo, pp. 176. Paris, 1826.

---

28. Traité d'Anatomie Topographique, ou Anatomie des Régions du Corps Humain, considérée spécialement dans ses Rapports avec la Chirurgie, et la Médécine Operatoire. Par Ph. Fréd. Blandin, Professeur particulier d'Anatomie, &c. 1 volume in 8vo, avec Atlas de Douze Planches, Dessinées sous le yeux de l'Auteur, par N. H. Jacob, Professeur de Dessin à l'Ecole Royale Veterinaire d'Alfort. Paris, 1826.

☞ *The letter-press contains a very excellent verbal description of the parts—while the plates present decidedly the most beautiful specimens of lithography we have ever seen. This is really a very meritorious production of Professor Blandin's.*

---

29. A short Inquiry into the principal Causes of the unsuccessful Termination of Extraction by the Cornea, with the View of shewing the Superiority of Dr. F. Jäger's Double Knife over the Single Cataract Knives of Wenzel and Beer. By Charles Loudon, Member of the Royal College of Surgeons in London. Quarto, with an Engraving, pp. 14. London, 1826.

☞ *Of all the operations proposed or practised for the removal of Cataract, extraction appears to be generally, if not invariably, the safest and most successful. This, we believe, is the opinion of the first ophthalmic surgeons of our country and our age. To diminish the difficulties and dangers attendant on this delicate operation, a new instrument of peculiar construction has lately been invented by Dr. Frederick Jäger, of Vienna. An introduction of this instrument to the notice of British surgeons constitutes the object of Mr. Loudon's concise but luminous inquiry. This gentleman, with an ardour for professional knowledge and distinction, highly honourable to his character, has passed three years in the medical schools of France, Germany and Italy, and paid, as this production evinces, great attention to ophthalmic surgery. It is written in a very plain, unostentatious style, and illustrated by a neat engraving of Dr. Jäger's knife.*

---

30. Meteorological Register for the Years 1822-3-4 and 5, from Observations made by the Surgeons of the Army, at the Military Posts of the United States. Prepared under the Direction of Joseph Lovell, M.D. Surgeon General of the United States' Army. Quarto, stitched, pp. 63. Washington, 1826.

---

31. Proceedings of the Phrenological Society of Washington, relative to two Lectures against the Science of Phrenology, delivered at the Columbian College by Dr. Thomas Sewall, Professor of Anatomy and Physiology, in May, 1826. Octavo, stitched, pp. 8.

---

32. Myology, illustrated by Plates of a peculiar Construction, in Four Parts. Part the Second, containing the Muscles on the Anterier and Posterior Parts of the Arm and Hand; and Part the Third, containing the

Muscles of the Abdomen and Back. By E. W. Tuson, Lecturer on Anatomy and Physiology, Member of the Royal College of Surgeons, late House-Surgeon to the Middlesex Hospital, London. Callow and Wilson, 1826. Price, 12s. plain, 18s. coloured, each part; or the Four Parts, in boards, 2l. 8s. plain, or 3l. 12s. coloured.

☞ *We can safely recommend these Plates of Mr. Tuson's to the notice of the student. The different layers of muscles, their origins and insertions, are ingeniously and accurately displayed, and that in a manner which must recall to the memory of the young anatomist, more vividly than common plates can do, the myological labours in the dissecting room. They also form an excellent prelude to a dissection.*

---

33. An Oration delivered on Thursday, February 9th, 1826, before the Hunterian Society: with Supplementary Observations and Engravings. By Sir William Blizzard, Knt. F.R.S. &c. 4to, pp. 40, with two Plates. Underwoods, 1826.

---

34. A Letter on the Medical Employment of White Mustard Seed. By a Member of the London College of Surgeons. 8vo, stitched, pp. 31. London, 1826.

---

35. Dissertatio Medica Inauguralis de Pleuræ Inflammatione. Auctore, Jacobo Eaton, M.D. Edinburghi, 1826.

---

36. A Treatise on the Diseases of Children; with Directions for the Management of Infants from the Birth. By the late Michael Underwood, M.D. Eighth Edition, revised, with Notes and Observations, by Samuel Merriman, M.D. F.L.S. &c. 8vo, pp. 634. London, Callow and Wilson, &c. 1827.

☞ *This, as might be expected from the character of the editor, is a greatly improved edition, not only on account of what is added, but of what is omitted.*

---

*** Note.—Authors and publishers will readily appreciate the importance of having their works recorded, with full length titles, on this list, which stands as a perpetual advertisement so long as the Journal lasts, and as far as it extends. The republication of the Journal in America enhances the advantages of the Bibliographical Record, on which no work can be entered, unless transmitted, free of expense, to the Editor, under sealed cover to the publishers, or in any other way most convenient to the parties concerned.

*N. B.—As the Bibliographical Record closes on the 15th of the month preceding publication, all Works received after that date necessarily stand over till next quarter.*

---

MEDICO-CHIRURGICAL REVIEW.

*(American Edition.)*

All the back Numbers of this Journal, from No. 1 to No. 16, inclusive, have now been imported (by permission of the Editor) by Mr. Highley, (now the Publisher of the Medico-Chirurgical Review) who can supply gentlemen with complete sets, according to order.

☞ *The American edition is printed verbatim from the London edition, and detached numbers will correspond so as to complete imperfect sets without any deformity to the work.*

174, *Fleet Street, December* 21*st*, 1826.

### *Additional Subscribers since last Quarter.*

Barry, Dr. David, Member of the Royal College of Physicians of London, &c. 7, Great Mary-la-bonne Street, Cavendish Sq.
Buchanan, Dr. William, Assistant Surgeon, Bengal Establishment
Churchill, Mr. Surg. Park Street, Grosvenor Square
Dalton, Mr. Surgeon, &c. High Street, Cheltenham
Embleton, Mr. Surg. Banborough
Ernests, Dr. Sheffield, Yorkshire
Fergusson, Dr. Windsor
Geirrard, Dr. Feversham
Heygate, Mr. James, Surgeon, Hamstope (we believe)
Huie, Richard, M. D. F. R. S. A. &c. George Square, Edinburgh
Lamont, Dr. J. B. Jamaica
Lewis, Mr. Surgeon, Member of the Royal College of Surgeons of London, Tenby, South Wales
Luscombe, Mr. Yealinton
M'Andrew, Dr. Assistant Surgeon of the 1st Foot—to Madras
M'Manus, Dr. Piazza St. Trineta, Florence
M'Manus, Mr. Edinburgh
Medical Society, Borough
Miller, Dr. 9, New Broad St. City
Owen, Mr. William, Member of the Royal College of Surgeons, and Licentiate of the Worshipful Society of Apothecaries, London, Manchester
Pretty, Mr. Surgeon, Mabledon Place, Russell Square
Ralph, Mr. Surgeon, Leicester Square
Simpson, Mr. W. Member of the Royal College of Surgeons of London, &c. Hammersmith
Spelling, Mr. Surgeon, Ecclesfield
Strachan, Dr. Calcutta
Watt, William C. Surgeon, R.N.
Wheeler, Mr. Surgeon, Bayswater
William, Mr. O. G. Surg. Swansea

